# Supervised brain node and network construction under voxel-level functional imaging

**DOI:** 10.1162/IMAG.a.56

**Published:** 2025-06-26

**Authors:** Wanwan Xu, Selena Wang, Simiao Gao, Xinyuan Tian, Chichun Tan, Xilin Shen, Wenjing Luo, Todd Constable, Tianxi Li, Yize Zhao

**Affiliations:** Department of Biostatistics, Yale School of Public Health, Yale University, New Haven, CT, United States; Department of Biostatistics, School of Public Health, Brown University, Providence, RI, United States; Department of Radiology and Biomedical Imaging, Yale School of Medicine, Yale University, New Haven, CT, United States; School of Statistics, University of Minnesota, Minneapolis, MN, United States

**Keywords:** brain atlas, connectome-based predictive model, fMRI, functional connectivity, spectral clustering, supervised learning

## Abstract

Recent advancements in understanding the brain’s functional organization related to behavior have been pivotal, particularly in the development of predictive models based on brain connectivity. A major analytical strategy in this domain involves a two-step process by first constructing a connectivity matrix from predefined brain regions, and then linking these connections to behaviors or clinical outcomes. Although some advances considered subject-specific functionally homogeneous nodes without relying on predefined regions of interest (ROIs), all these approaches with unsupervised node partitions predict outcomes inefficiently with independently established connectivity. In this paper, we introduce the Supervised Brain Parcellation (SBP), a brain node parcellation scheme informed by the downstream predictive task. With voxel-level functional time courses generated under resting-state or cognitive tasks as input, our approach clusters voxels into nodes in a manner that maximizes the correlation between inter-node connections and the behavioral outcome, while also accommodating intra-node homogeneity. We rigorously evaluate the SBP approach using resting-state and task-based fMRI data from both the Adolescent Brain Cognitive Development (ABCD) study and the Human Connectome Project (HCP). Our analyses show that SBP significantly improves out-of-sample connectome-based predictive performance compared to conventional step-wise methods under various brain atlases. This advancement holds promise for enhancing our understanding of brain functional architectures with behavior and establishing more informative network neuromarkers for clinical applications.

## 1 Introduction

Understanding brain functional organization through large-scale networks and how such topology relates to behavior are two of the fundamental themes in neuroscience. By partitioning the brain into a collection of regions or nodes, whole-brain functional connectivity or connectome can be constructed using functional magnetic resonance imaging (fMRI) under a resting state or different cognitive tasks to characterize functional dependence, encapsulated within network configurations spanning across all the nodes. Subsequently, the established functional connectivity can serve as predictive entities for a set of learning methods called connectome-based predictive model (CPM) (Shen et al., 2017), and have been linked with normal cognitive processes and a variety of disorders such as anxiety (Z. Wang et al., 2021), obsessive-compulsive disorder (Wu et al., 2023), and Parkinson’s disease (X. Wang et al., 2022).

To establish functional connectivity, it is essential to introduce a brain parcellation scheme to define nodes, upon which functional connections are built. Over recent decades, extensive efforts have been devoted to constructing brain parcellations that reflect neuroanatomical or functional organizations, offering insights into behavioral outcomes such as seizures, sclerosis lesions, cerebrovascular diseases, and other neurological disorders (Nowinski & Chua, 2013;Revell et al., 2022;Shen et al., 2013;Shiee et al., 2010). Initial parcellation efforts created atlases based on brain cytoarchitecture or anatomical configurations, including the Brodmann-based automatic anatomic labeling atlas (AAL) (Amunts & Zilles, 2015;Brodmann, 1909;Tzourio-Mazoyer et al., 2002). However, such anatomical atlases often contained regions too coarse for effective functional connectivity studies. Alternatively, fMRI-based parcellations emerged under the premise that functional signals within a node should exhibit coherence. Fine-scale functional parcellations have been achieved by identifying homogeneous modules using graph-based models or boundary detection techniques (Craddock et al., 2012;Fan et al., 2016;Gordon et al., 2016;Shen et al., 2013;Thomas Yeo et al., 2011). For example,Glasser et al. (2016)developed a multi-modal parcellation of the human cerebral cortex through a two-step process: first, generating group-level neuroanatomical parcellations with potential areal borders, followed by training a machine-learning classifier to identify cortical areas in individual subjects. Other studies (Fan et al., 2021;Ji et al., 2009) directly analyzed raw fMRI time series using signal separation models, such as independent component analysis (ICA), to uncover functional alignment among time series. Moving forward along this line, recent studies indicated reconfigurations of brain functional architectures with sex, cognitive states, and task demands (Cole et al., 2014;Salehi et al., 2018;Yeo et al., 2015). This growing body of research suggests that functional regions are dynamic in their boundary definitions, advocating for a flexible atlas that adapts to specific functional involvements. Another line of research aims to understand variability in brain networks across individuals while ensuring findings remain generalizable to larger populations. To this end, recent studies have employed group-level ICA to decompose fMRI signals into consistent functional networks shared across subjects. For example, recent work leveraged resting-state fMRI data from over 100,000 individuals across private and public datasets to identify robust multi-spatial-scale intrinsic connectivity network (ICN) templates using multi-model-order ICA (Iraji et al., 2023). Further advancements have extended these methods to capture within-subject variability, including in a time-resolved manner (Boukhdhir et al., 2021;Iraji et al., 2023;Nurmi et al., 2024). In addition, several approaches focus on individual-specific brain parcellations, such asD. Wang et al. (2015),Kong et al. (2021), andChong et al. (2017), which aim to capture fine-grained, subject-level brain organization.

**Fig. 1.**
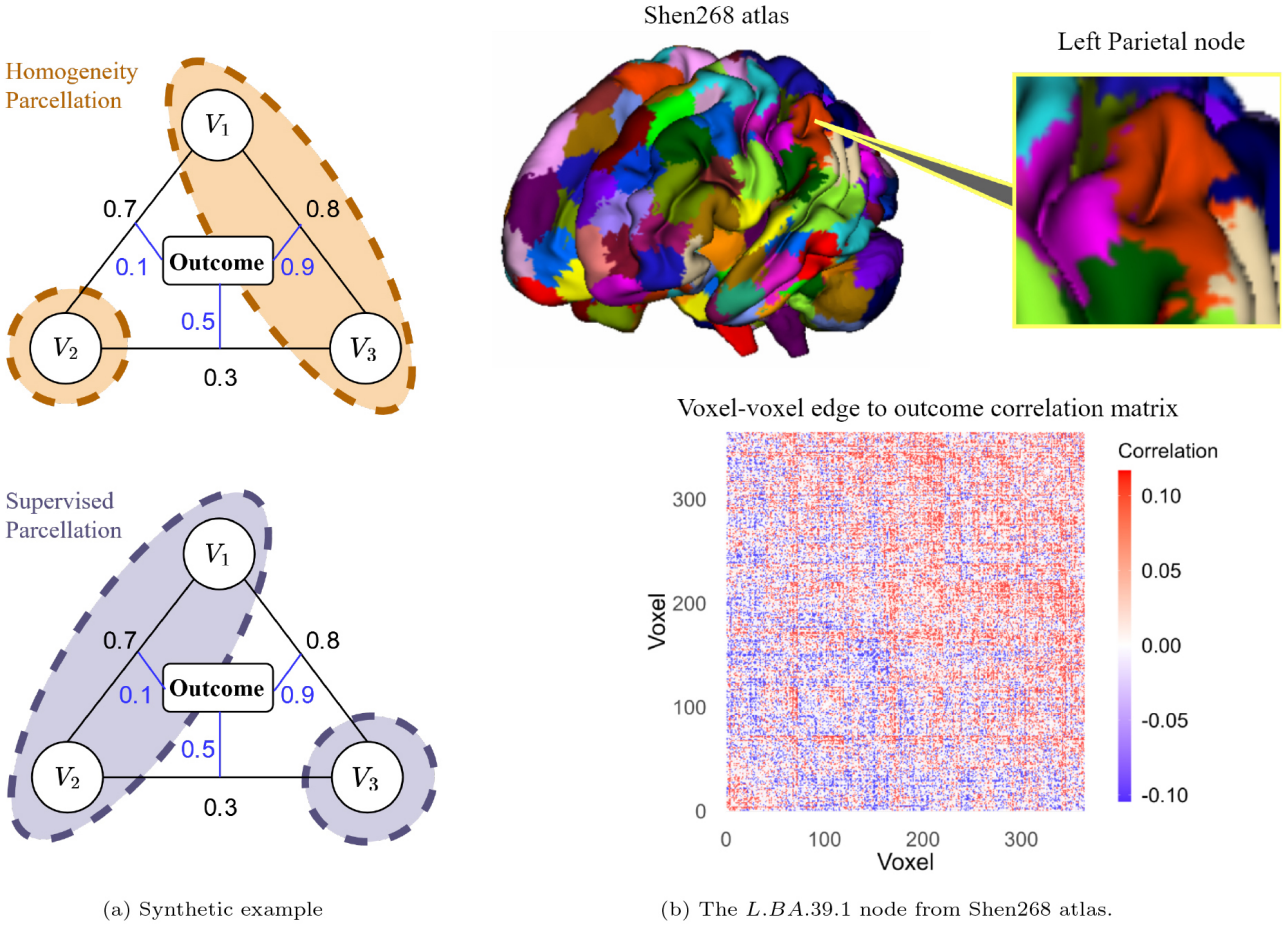
(a) Illustration of the proposed method using a synthetic example with three voxels labeled$V_{1}$,$V_{2}$, and$V_{3}$, represented by circles. Above black lines, numerical values indicate the strength of the functional connection between voxel pairs, while next to blue lines, values denote the strength of the association between each voxel-level connection and the target outcome. The top panel displays the existing regional homogeneity parcellation scheme with orange ellipses, and the bottom panel illustrates the proposed supervised parcellation with blue ellipses. (b) A detailed view of node 184 (left parietal area) from the Shen268 atlas (Shen et al., 2013), analyzed during the MID task from the ABCD study. Functional connections are quantified by the Pearson correlation of the time series, with only the top25%absolute values displayed in the heatmap. Positive correlations are shown in red, and negative correlations are in blue, providing a visual contrast for the cognitive composite score association.

Conversely, in efforts to establish brain-to-behavior correspondence and develop predictive models under connectivity data, existing unsupervised parcellation schemes that aim for a marginal uniformity within individual regions may lose their relevance and can even become counter-productive. This is because when computing a functional connection between two nodes, the initial step involves averaging all voxel-level time series within a node. Subsequently, the statistical dependency is calculated between each pair of the averaged time courses. In this process, all the connectivity signals within a node are fully saturated, overlooking the potential intra-region functional heterogeneity linked with the target outcome (Luo & Constable, 2022). To elucidate this concept,Figure 1apresents a synthetic example with three voxels,$V_{1},V_{2},V_{3}$, represented as circles, assuming that the functional edges between each pair of voxels are 0.8, 0.7, and 0.3, as illustrated, and that their associations with the outcome are 0.9, 0.1, and 0.5, respectively. Given the high similarity in voxel-wise functional signals between$V_{1}$and$V_{3}$indicated by a large correlation, it is expected that these two voxels would be grouped into the same node, separate from$V_{2}$under an existing homogeneity-focused parcellation scheme. However, this overlooks the highly predictive edge between them, leading to an average node-wise connectivity-to-outcome correlation of only 0.3. In contrast, when the downstream learning objective is considered during the parcellation learning, node definitions should ideally reveal the strongest predictive edges. As depicted in the lower panel ofFigure 1a, a supervised parcellation scheme would group$V_{1}$and$V_{2}$by keeping the highly predictive edges as inter-node ones. Such a parcellation yields an average correlation of 0.7 between node-wise functional connections and the outcome, demonstrating its potential effectiveness.

To this end, we introduce a novel Supervised Brain Parcellation (SBP) scheme that leverages the relationship between state-specific functional connectivity alterations and behavioral trait variations. We posit that the delineation of functional boundaries for node construction should not only reflect anatomical structure but also enhance the predictive power of network architectures and reliably identify functional neuromarkers. This is essentially different from most of the existing parcellation methods that only prioritize functional homogeneity or anatomical organization while neglecting how inter-regional connectivity relates to behavioral variability. Specifically, our proposed supervised parcellation learning framework, inspired by regularized spectral clustering, takes into account the correspondence between the defined functional connections and the behavior outcome by jointly optimizing functional connectivity and behavioral relevance, producing brain regions that are both functionally coherent and predictive of behavior. This trait-specific approach is considerably more adaptable to downstream learning tasks, and it operates independently in both training and testing phases. Meanwhile, in contrast to existing methods such as multivariate pattern analysis (MVPA) (Haxby, 2012), which predict behavior using pre-defined features or voxel-wise patterns, SBP promotes spatial contiguity while explicitly modeling behaviorally relevant functional connections. InSuppmentary Figure 3, we show that SBP provides finer parcellation in node 184 following the previous demonstration example. Through the analyses of two landmark studies, the Adolescent Brain Cognitive Development (ABCD) study and the Human Connectome Project (HCP) under their resting-state and task-based fMRI data, we demonstrate that voxel-to-node memberships are reconfigured under different cognitive conditions with the proposed supervised parcellations substantially enhancing the out-of-sample predictive performance for general intelligence compared with the existing parcellation methods. This work contributes to the evolving paradigm of brain parcel constructions by acknowledging that functional brain regions are not confined to static boundaries that are invariant to different downstream tasks, but could be considered with adaptability and responsiveness to their functional engagement. It illuminates the path toward objective-enhanced and information-supervised parcellations, with the resulting functional organizations providing significant benefits for establishing brain-to-behavior learning models and uncovering neurobiological network signals.

The remaining of this paper is organized as follows: InSection 2, we introduce our experimental studies, provide empirical evidence on signal obscuration, detail our supervised parcellation learning framework, and describe the optimization algorithm and model evaluation procedures. InSection 3, we apply the model to create parcellations using fMRI under varying cognitive conditions and behavioral data from the ABCD and HCP, and conduct independent validation and testing to evaluate predictive and replicative efficacy. The paper concludes with a discussion inSection 4.

## 2 Material and Methods

### 2.1 Data

To demonstrate our method and show the power of the developed functional parcellations, we utilize resting-state and task-based fMRI and behavioral data collected for each subject from the ABCD and HCP studies. We also demonstrate the motivation for this work through a data example extracted from the ABCD study and illustrate the core idea of our method with a simulated dataset.

#### 2.1.1 ABCD study

The ABCD study is an ongoing prospective study launched in 2015 to investigate brain development and adolescent health for more than 10,000 children aged 9 to 10 years from 21 sites across the United States (Garavan et al., 2018). The study has been collecting a wealth of measures on brain imaging, biospecimens, and cognitive development measurements to support different dimensions of brain-to-behavior studies. For the fMRI images, each participant went through a scan session in a fixed order beginning with a localizer, acquisition of 3D T-weighted images, 2 runs of resting-state fMRI, T2-weighted images, 1-2 runs of resting-state fMRI and task-based fMRI. The ABCD study collected three task-based fMRI including an emotional version of the n-back task (nback) (Cohen et al., 2016)), the Monetary Incentive Delay (MID) task (Knutson et al., 2000), and the Stop Signal task (SST) (Logan, 1994). These task domains involve cognitive functions related to working memory, emotion regulation, reward processing, motivation, impulsivity, and impulse control (Casey et al., 2018). More details on the imaging acquisition across different sites and pre-processing are described elsewhere byGreene et al. (2018);Casey et al. (2018); andHagler Jr et al. (2019).

We focused on the first release of fMRI data (Released 3.0.1). Raw DICOM images for5772subjects were collected via ABCD fast track (Casey et al., 2018) and preprocessed using BioImageSuite (Papademetris et al., 2006). All fMRI images were realigned to correct for motion and registered to the standardized3 mm×3 mm×3 mmcommon space. Only subjects with low-motion fMRI data (mean frame-to-frame displacement<0.15 mmand maximum frame-to-frame displacement<2 mm) at3 mmresolution were included in our analyses. We are interested in the total cognition composite score from the NIH Toolbox Cognition Function Battery (Akshoomoff et al., 2013;Heaton et al., 2014) as the target outcome. This score encapsulates two cognitive abilities: “crystallized,” based on past learning, and “fluid,” indicative of novel learning and information processing in unfamiliar contexts. We employed the total cognition composite score for its demonstrated test-retest reliability and its high correlation with cognitive summary scores in the literature (Heaton et al., 2014). After preprocessing,1589subjects, consisting of858(54%) female and731(46%) male, were included in the study. The input images have dimensions of61×73×61×396, where the first three axes represent voxel coordinates, and the last represents time points. To enable comparisons with existing atlases, only54,971voxels included in the AAL atlas (Rolls et al., 2020) were retained. Voxels with only zero time courses were treated as missing data. We computed the missing rate for each subject under each functional condition (Rest, MID, nBack) and excluded the top5%of subjects with the most missing voxels. For the remaining1509subjects, the overlapping voxels with complete time courses were included in the model. Finally, we constructed the voxel-level connectivity maps for each subject under each condition using Pearson correlation coefficients for the time courses between each pair of voxels.

#### 2.1.2 HCP

We also independently evaluated our method using the HCP study to show the robustness of the predictive performance. The HCP aims to map macroscopic human brain circuits and their behavioral correlates in a large population of healthy adults. We utilized a subset of the HCP S900 release with subjects who have collected voxel-level fMRI data for all nine functional sessions, which included two resting-states and seven task-based scans. Subjects with excessive head motion, defined as a mean frame-to-frame displacement exceeding0.1 mmor a maximum frame-to-frame displacement exceeding0.15 mm, were excluded from the analysis. Eventually, the analyses focused on a dataset of494subjects. The preprocessing followed the same procedures asLuo et al. (2021)andSalehi, Greene, et al. (2020), using the HCP minimal preprocessing pipeline (Glasser et al., 2013) and subsequent processing with BioimageSuite (Joshi et al., 2011). Consistent with the ABCD study, we limited the analysis to the54,971voxels within the AAL atlas.

Notably, the motion exclusion criteria were intentionally adjusted between the ABCD and HCP datasets to account for differences in participant demographics and data characteristics. Given that the ABCD study consists of preadolescent children, who generally exhibit higher motion levels, a slightly more relaxed threshold was applied to retain a sufficient number of high-quality scans while minimizing data loss. In contrast, the HCP dataset consists of young adults with relatively lower motion tendencies; and we applied a slightly stricter motion exclusion threshold to ensure optimal data quality. The ABCD and HCP datasets differ significantly in terms of acquisition protocols, age groups, and overall motion characteristics. Our primary goal is to demonstrate the generalizability of the proposed SBP method across different datasets rather than to enforce identical results. By tailoring motion exclusion thresholds to each dataset’s unique characteristics, we ensure a more balanced comparison and validation of SBP across datasets.

After removing missingness, the common voxels with complete time courses across the469subjects were used. Similarly, we summarized a voxel-level functional connectivity matrix for each subject and each state as our input. For the behavior outcome, we consider the fluid intelligence score assessed using a form of Raven’s progressive matrices with 24 items (Bilker et al., 2012).

### 2.2 Signal obscuration

We first illustrate how current brain atlases could obscure crucial intra-node signals in functional connectivity analysis. Using data extracted from the ABCD study as an example, we focus on functional connectivity constructed under the MID task with nodes defined by a commonly used Shen atlas (Shen et al., 2013) with 268 nodes. Particularly, we take the node 184 located in the left parietal area (Brodmann area 39) as a case study, exemplified inFigure 1b. Within this node, there are364voxels, which together form66,066intra-node voxel-level functional edges. When correlating each functional edge with the cognition composite score, we obtain a wide range of correlation strengths, from$0.80\times 10^{-6}$to0.12, with44.42%showing negative correlations and55.58%positive. The diverse range of values within a single node indicates the heterogeneity in how different parts of the node relate to cognitive behavior; and we can also observe these spreading signals in the heatmap represented inFigure 1b. When further examining functional connections between node 184 and other nodes, the average significant correlation between functional connections and the cognition composite score is only 0.01, which is considerably lower than what might be expected from a more refined intra-node connectivity. This highlights an opportunity to enhance functional network predictive accuracy with a more informative parcellation scheme.

### 2.3 Supervised brain parcellation

Suppose that under a cognitive state, we have fMRI scans collected forpvoxels across the whole brain for each of thensubjects. The behavior outcome of interest is measured and denoted as$\text{o}=\left(o^{\left(1\right)},o^{\left(2\right)},\ldots ,o^{\left(n\right)}\right)\in {\mathbb{R}}^{n}$with$o^{\left(i\right)}$representing the outcome for subjecti. The general objective is to partition thepvoxels intoKgroups, denoted by the voxel index sets${\left\{C_{k}\right\}}_{k=1}^{K}$; and we require$C_{k}\cap C_{k^{\prime }}=\emptyset$for$k\neq k^{\prime }$and$\cup_{k=1}^{K}\;C_{k}\;=\left\{1,2,\ldots ,p\right\}.$By treating each group as a region/node, we can then establish node-level functional connectivity as neuromarkers to predict behavior outcomes.

One way to tackle this brain partition problem is through graph community detection by constructing a weighted indirect graphG=(V,E,W). The vertex setVconsists of all the voxels, and edge setEincludes all connections under weightsW. We consider the statistical dependence of the functional time courses as the weight metric, reflecting the functional organization. The graphGcan be uniquely represented by an adjacency matrix$A^{\left(i\right)}=\left(a_{jl}^{\left(i\right)}\right)\in {\mathbb{R}}^{p\times p}$for subjecti, which also serves as a voxel-level functional connectivity matrix. Our objective here is to find a brain parcellation$\cup_{k=1}^{K}\;C_{k}$that is shared among subjects. Despite some attempts at an individual brain parcellation (Salehi, Karbasi, et al., 2020), our study aims for a parcellation that can be directly generalized to independent samples for predicting behavioral outcomes, making a groupwise parcellation structure ideal. To aggregate adjacency matrices${\left\{A^{\left(i\right)}\right\}}_{i=1}^{n}$across samples and form a groupwise adjacency matrixA, we consider the following options:

$$\begin{array}{l} 1)\;A=\frac{1}{n}\sum_{i=1}^{n}A^{\left(i\right)}⊙A^{\left(i\right)};\;\;2)\;A=\frac{1}{n}\sum_{i=1}^{n}A^{\left(i\right)};\;\\ 3)\;A=\frac{1}{n}\left\{\sum_{i=1}^{n}A^{\left(i\right)}⊙A^{\left(i\right)}-D^{\left(i\right)}\right\}. \end{array}$$ (1)

Here,⊙represents the Hadamard product, and$D^{\left(i\right)}$is the diagonal matrix called degree matrix with the row sum of$A^{\left(i\right)}$as diagonal elements. Among those options, the first one is supported by rigorous statistical justifications that topological structures could be incorporated in the mean squared connectivity matrix (Lei & Lin, 2022); the second one represents a commonly adopted sample mean matrix for a group; and the final one is a refined version for option one with rescaling to stabilize the algorithm. In our numerical studies, all three options are considered during implementation, and we observe similar performance among these realizations indicating a robustness of our numerical operations for the group-level adjacency information among voxels.

If we only focus onAto perform community detection without consideration of the downstream predictive task, canonical spectral clustering is a powerful approach and has been widely used for constructing unsupervised brain parcellations (Kim et al., 2010;Shen et al., 2013). This method typically utilizes the top eigenvectors of either network adjacency matrix or Laplacian matrix to segment the graph into distinct communities, leveraging spectral characteristics (Lei & Lin, 2022). Through such a learning procedure, we ensure that the connections between different communities exhibit low similarity, whereas those within the same community demonstrate high similarity. To further incorporate correspondence with the behavior outcome of interest when constructing functional nodes, on top of the adjacency matrixA, we create a separation preference matrix$R=\left(r_{jl}\right)\in {\mathbb{R}}^{p\times p}$with the outcome information involved as a “guidance” to learn the boundary of partitions. The goal of the constructed preference matrix is to leverage the predictive power of the functional connection between voxeljand voxellto regularize the spectral learning procedure. For each entry$r_{jl}$in the preference matrix, we use the Pearson correlation between${\left\{a_{jl}^{\left(i\right)}\right\}}_{i=1}^{n}$and${\left\{o^{\left(i\right)}\right\}}_{i=1}^{n}$as a natural choice to characterize the association between the edge and the outcome. One can also use the inverse pvalue of the Pearson correlation to regularize under statistical significance. Combining two sides of information, the proposed SBP can be formulated as the following optimization procedure,

$$\begin{array}{cc} \underset{{\left\{C_{k},\mu_{k}\right\}}_{k=1}^{K}}{\text{min}} & \sum_{k=1}^{K}\left(\sum_{j\in C_{k}}∥U_{j\cdot }-\mu_{k}{∥}^{2}+\lambda \sum_{j,l\in C_{k}}r_{jl}\right),\\ \text{such that} & C_{k}\subset \left[p\right],\;\;\;\;\;C_{k}\cap C_{k^{\prime }}=\emptyset ,\overset{\;\;\;\;\;\;K}{\underset{k=1}{\cup }}C_{k}=\left[p\right]. \end{array}$$ (2)

Here$U_{j\cdot }$denotes thej-th row of matrix$U\in {\mathbb{R}}^{p\times K}$withj=1,…,p, where columns ofUare theKleading eigenvectors ofAcorresponding to the highest absolute eigenvalues; and$\mu_{k}=\sum_{j\in C_{k}}U_{j\cdot }/\left|C_{k}\right|$taking the mean across rows$U_{j\cdot }$for each$j\in C_{k}$standardized by the cardinality of set$C_{k}$denoted as$\left|C_{k}\right|$. Optimization (2) indicates that our main loss function consists of two components. The first component uses the groupwise functional connectivity adjacency matrix to guide the clustering through spectral learning. This part attempts to assign each voxel to the nearest centroid with the smallest Euclidean distance, which respects the pattern of the sample connectivity maps. The second component incorporates the influence of behavioral outcomes to refine the learning process. By introducing the regularization matrixR, we impose penalization to each of the entries$\left\{r_{jl}\right\}$withj,lbelonging to the same node. By using the edge-to-outcome correlation values as entries in this matrix, we effectively regularize the stronger connections during the minimization process. This strategy is the key to ensure that the most informative signals are not lost. It leads to the clustering of voxels with highly predictive connections into distinct nodes, thereby preserving the integrity and predictive power of important functional connections. Within the optimization, the second component is controlled by a tuning parameterλ. Ifλ=0, the model reduces to a standard spectral clustering of adjacency matrixA, as described inLei and Lin (2022). To determine the optimal valueλ, we employ the cross-validation method. The detailed procedure and considerations are explained inSection 2.3.2. In essence, our learning strategy encompasses two key aspects: the similarity of connectivity patterns within nodes and the effective distribution of voxels. The latter involves grouping voxels linked by highly predictive connections into different nodes to preserve their predictive power. InFigure 2, we demonstrate a brief workflow of the proposed SBP.

**Fig. 2.**
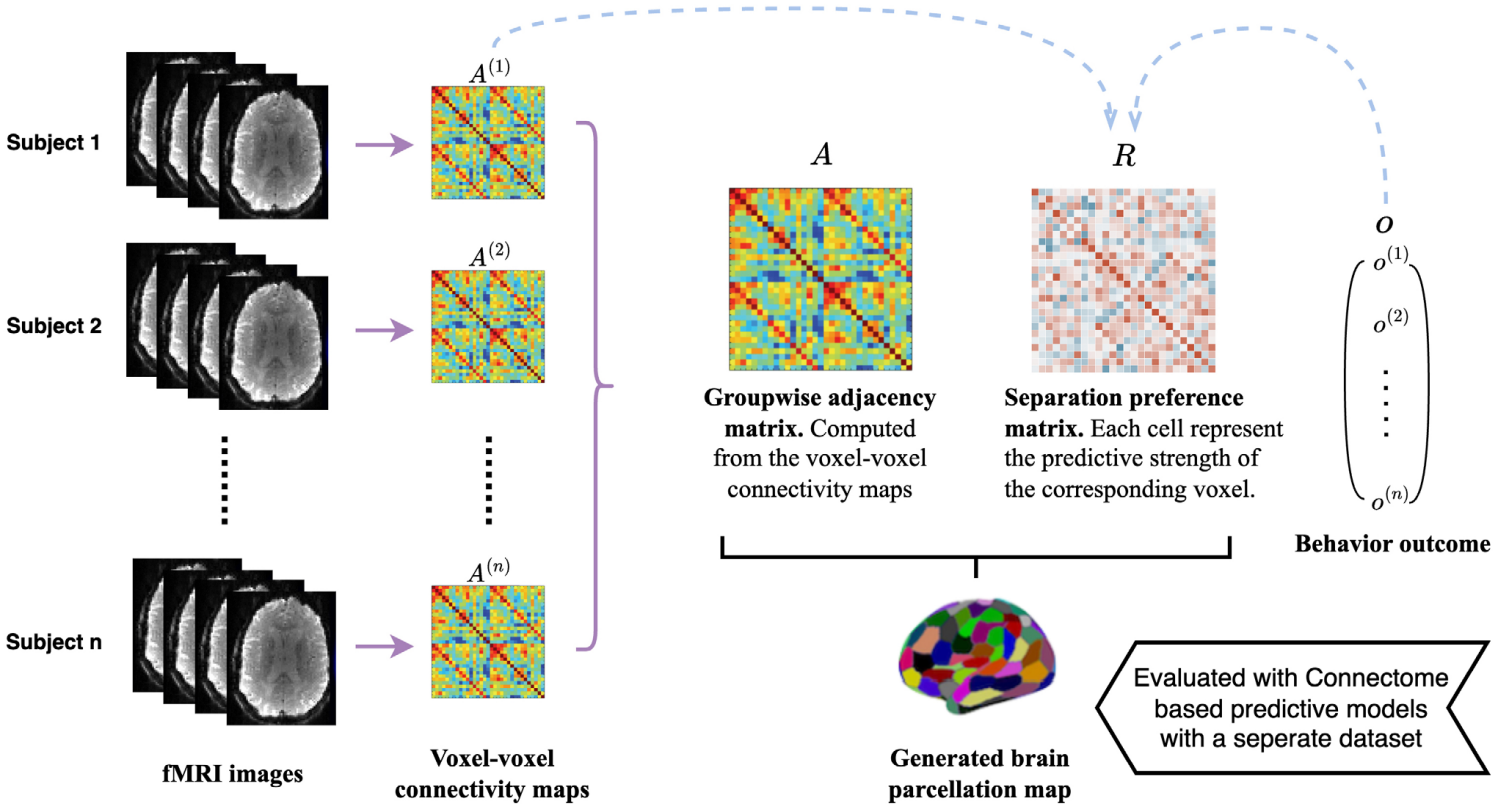
Flowchart of the proposed Supervised Brain Parcellation (SBP) scheme with both fMRI images and a behavior outcome.

#### 2.3.1 Coordinate descent algorithm on solving SBP

Given our optimization problem is non-convex, we employ an efficient coordinate descent algorithm to conduct the minimization for (2). This method involves iterative updates on both voxel assignments and cluster mean values. The process begins by initializing the parcellation set$\left\{C_{k}\right\}$. In practice, we could start with the results from standard K-means clustering on the rows of matrixU. In each iteration, for voxelj∈{1,…,p}, the new cluster label$k^{j}$is determined as the one that minimizes the following objective function:

$$k^{j}={\text{argmin}}_{k=1,\ldots ,K}\left\{-2U_{j\cdot }^{T}\mu_{k}+∥\mu_{k}{∥}^{2}+\lambda \sum_{l\in C_{k}}r_{jl}\right\}.$$

This function considers both the distance of the voxel to the cluster centroids and the separation preference matrix to jointly guide the cluster updates. Subsequently, the centroid of each cluster is recalculated as the mean of rows inUcorresponding to the newly assigned group labels in that cluster. We then perform a convergence check, assessing whether the change in the loss function is below a set tolerance level ($10^{-5}$in our case). In Algorithm 1, we summarize each step in the SBP algorithm, which can be seen as a regularized adaptation of Lloyd’s algorithm (Sabin & Gray, 1986). Of note, similar to the standard K-means problem, our objective is non-convex, and there is no guarantee of convergence to the global optimum. To mitigate this, we suggest multiple runs of the algorithm with varied initializations. The iteration yielding the smallest objective value is chosen as the final solution. In our numerical studies, we extensively evaluate the algorithm’s performance and generally observe its effectiveness in converging towards a global solution.

**Table:** 

**Algorithm 1: Supervised Brain Parcellation (SBP)**
**Input:** - ${\left\{A^{\left(i\right)}\right\}}_{i=1}^{n}$ : voxel-level connectivity matrices for n subjects, - R : separation preference matrix, - K : number of nodes, - λ: tuning parameter. **Procedure:** 1. Construct groupwise adjacency matrix A . 2. Perform eigendecomposition on A . Select the leading K eigenvectors based on absolute eigenvalues. Let U be the matrix with these eigenvectors as columns. 3. Initialize the cluster assignments $C_{1},\ldots ,C_{K}$ . Compute cluster cardinality $\left|C_{k}\right|$ and centroid $\mu_{k}=\sum_{j\in C_{k}}U_{j.}/\left|C_{k}\right|$ for each k=1,…,K . 4. Repeat the following steps until converged or max iterations are reached: (a) Update voxel assignment: For each voxel j=1, 2,…,p , compute the loss for assigning it to the k -th node: $∥U_{j\cdot }-\mu_{k}{∥}^{2}+\lambda \sum_{l\in C_{k}}R_{jl}$ . Find the node $k^{j}$ with the smallest loss. Assign voxel j to node $C_{k^{j}}$ . (b) Update node centroid: For each node k=1, 2,…,K , compute the number of voxels assigned $\left|C_{k}\right|$ , and update the k -th centroid to the corresponding mean of rows $\mu_{k}=\sum_{i\in C_{k}}U_{i.}/\left|C_{k}\right|$ . (c) Check for convergence: If the centroids do not change or the change is smaller than the tolerance level, convergence is reached, exit loop. **Output:** - ${\left\{C_{k}\right\}}_{k=1}^{K}$ : assigned index sets, k=1, 2,…,K ; - ${\left\{\mu_{k}\right\}}_{k=1}^{K}$ : node centroids, k=1, 2,…,K .

#### 2.3.2 Hyperparameter selection

To implement the algorithm to construct SBP, we consider a range of node numbersKin our numerical studies, varying from 100 to 500 in light of most of the existing brain parcellation sizes. Consistent with previous studies (Salehi et al., 2018;Shen et al., 2013), our goal is not to identify the optimal number of nodes for SBP as such an optimum may not exist. Instead, we focus on evaluating the model performance across various configurations of parcellation sizes. Regarding the tuning parameterλwhich balances the contribution to the parcellation construction between the spectral configuration of brain functional organizations and their induced predictive effect, we determine its value through a process involving training, validation, and testing. For each dataset$\left(A^{\left(i\right)},\;o^{\left(i\right)},\;i=1,\;\ldots \;,\;n\right)$, we randomly divide it into 10 folds with one-fold set aside as a testing set, while the remaining 90% samples are further randomly split into 10 folds for a cross-validation to determineλ. Under each split, 9 folds serve as training and 1 fold as validation. With a fixedλ, the SBP is trained on each training set to determine the node assignment${\left\{C_{k}\right\}}_{k=1}^{K}$to form a brain parcellation. The generated parcellation is then applied to the validation set to construct the regional functional connectivity based on the averaged node-level functional time course. Subsequently, a connectome-based predictive model (CPM) (Shen et al., 2017) is utilized to link the constructed connectivity matrix with the behavior outcome under this validation set with the predictive R-square obtained. The validation prediction under the current parcellation is then summarized by averaging the R-square across all 10 validation sets. Finally, we apply the parcellation with the optimalλfrom the validation to the testing set to construct regional functional connectivity. The predictive power is then assessed by CPM with the testing set R-square recorded. By repeating the above steps for each fold as a test set, we obtain the final averaged out-of-sample R-square. Of note, the Separation Preference Matrix (R) is constructed exclusively using the training data in each fold of the cross-validation process without incorporating any information from the test set. Thus,Ris not fixed across all tests for each dataset, but is recomputed for each fold of the cross-validation using only the training data. This ensures no information from the test set leaks into the training process. Throughout the procedure, we maintain independence between the construction of the parcellation and the final predictive evaluation by separating the tuning parameter’s determination from the testing samples. This ensures that the testing phase remains unbiased and the results are reliable.

#### 2.3.3 CPM

Connectome-based Predictive Modeling (CPM) is a data-driven framework for developing predictive models that link brain connectivity patterns to individual behavioral outcomes using data from neuroimaging modalities like resting-state and task-based fMRI (Shen et al., 2017). The CPM process includes several key steps: feature selection to identify brain connections associated with behavioral measures, feature summarization to condense these connections into a single value per subject, model building using linear regression or similar methods, and cross-validation to assess model generalizability. Additionally, permutation testing evaluates the significance of the model’s predictions, while visualization techniques, such as glass brain plots or circle plots, help interpret the results. By emphasizing prediction and generalizability, CPM provides a robust and comprehensive approach to understanding brain-behavior relationships, ensuring that the identified associations are reliable across different datasets and populations.

### 2.4 Reproducibility

To ensure the reliability of our constructed SBP scheme, we evaluate its reproducibility among different runs. For the evaluation metrics, we adopt both the Dice coefficient and adjusted random index (ARI) to quantify the similarity among brain atlases that are generated from different runs of the model. For within-study evaluations, we assess reproducibility for tasks and resting states under both ABCD and HCP studies. For each state, with post-preprocessingnsubjects, we randomly select75%of the subjects 20 times to form a random sample set. For each sample set and eachK=200, 300, 400, the SBP algorithm is applied with the previously tuned optimal$\lambda^{*}$forK, as detailed inSection 2.3.2. We denote the resulting parcellation sets as$\left\{C^{q},\;q=1,\;2,\;\ldots \;,\;20\right\}$, where each$C^{q}=\left\{C_{1}^{q},\;\ldots \;,\;C_{K}^{q}\right\}$represents the partition results amongpvoxels for theqsample set. To align different parcellations to assess reproducibility, we then locate the most similar node from the remaining parcellations. For instance, for$C_{k}^{1}$, thek-th cluster from the first sample, we compute the metric with all other nodes from each of$\left\{C^{2},\;\ldots \;,\;C^{20}\right\}$. The one with the highest consistency metric is identified as the closest match, and the value are recorded asD(k, 1, q). The reproducibility for thek-th node from the current parcellation is then computed as the average of these 19 Dice coefficients or ARI, denoted$\bar{D}\left(k,1\right)=\frac{1}{19}\sum_{q=2}^{20}D\left(k,\;1,\;q\right)$. The overall reproducibility score for each node is weighted by its size relative to the total number of voxels. Following a similar procedure, we also measure the agreement between parcellations established under the two studies, and we focus on the resting state to mitigate the state difference.

## 3 Results

### 3.1 Simulated data

We first demonstrate the proposed methods against standard spectral clustering using a simulated binary graph. This graph comprises 100 voxels arranged in a10×10lattice with the corresponding adjacency matrix$A\in {\mathbb{R}}^{100\times 100}$. Based on the lattice, we have$A_{ij}=1$if voxeliandjare connected, and0otherwise. Simultaneously, we also define a separation preference matrix$R\in {\mathbb{R}}^{100\times 100}$as follows—we set$r_{ij}$to be a large value wheni∈(1, 2, … , 10)andj∈(11, 12, … , 20); and we also set a large$r_{i,i+1}$whenitakes from the set(8, 18, 28, … , 98). The remaining elements ofRare set to0. These non-zero entries in matrixRdivide the voxels into distinct groups while maintaining spatial contiguity. Ideally, we want to prevent merging the voxels with a nonzero$r_{ij}$into the same parcel to retain the highly predictive connectivity signals.

We apply the proposed SBP algorithm on the simulated data withλ∈{0, 5, 10}, and the resulting parcellations are displayed inFigure 3. In the figure, voxels grouped in the same parcel are labeled with the same number and different clusters are distinguished by colors. Whenλ=0, the proposed model reduces to standard spectral clustering marginally onA, focusing solely on spatial proximity’s spectral information and neglecting the predictive influence of the network on the outcome. Asλincreases, the SBP method effectively segregates to maintain the predictive relevance of the constructed edges, while still achieving spatially coherent clustering. Whenλ=5, clusters 6, 7, 8, 11, and 14 contain voxels that are not directly connected. Such a pattern is also observed for clusters 3, 4, 5, 8, 12, and 15 whenλ=10. These results are anticipated given that the penalty parameter associated with the separation-enforced term introduces a trade-off between the marginal pattern within the adjacency matrix and the predictive power linked with the targeted outcome. Compared to standard spectral clustering, SBP demonstrates a more nuanced approach, balancing spatial patterns with prediction-driven network separation.

**Fig. 3.**
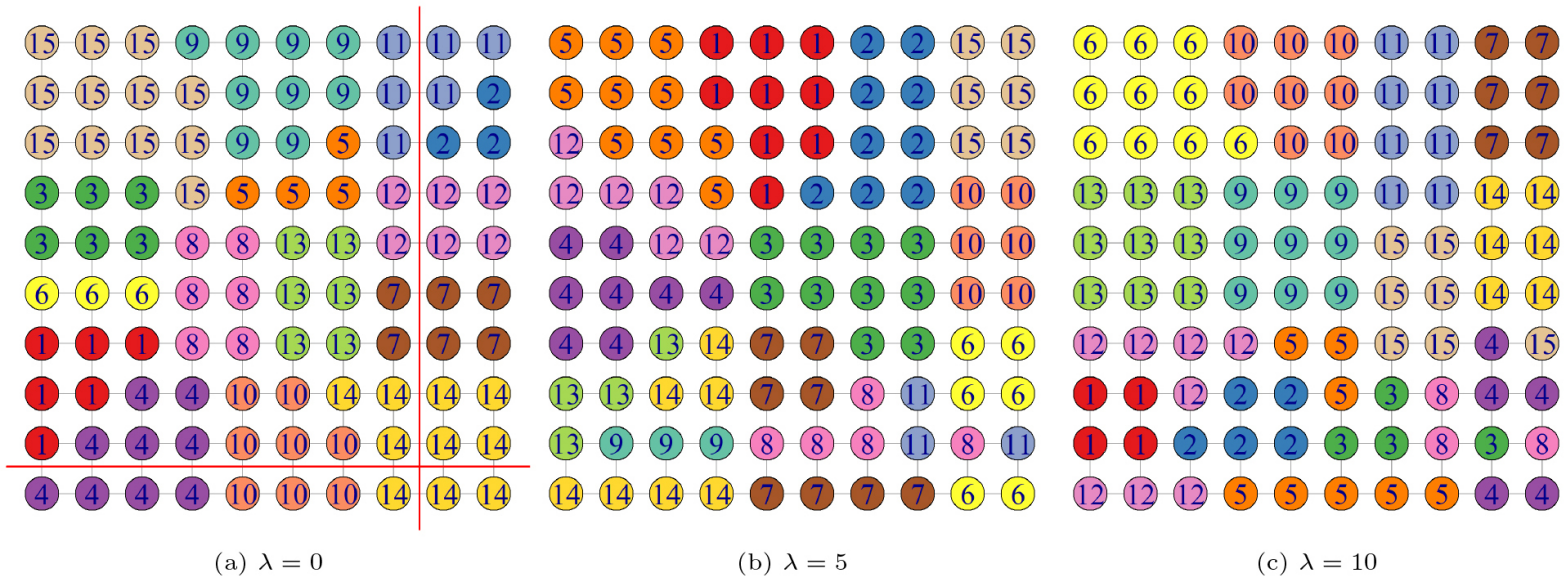
Demonstration of SBP with differentλvalues under simulated data. The nodes and edges reflect adjacency matrixA. The voxels cluster into the same parcel are assigned the same label and visualized by the same color. The red line segments indicate the high$R_{ij}$pairs that we ought to prevent from merging.

### 3.2 Predictive performance

We assess the connectome-based prediction for the cognitive scores based on the proposed SBP as compared to the existing parcellation schemes. We implement the proposed SBP withK∈{100, 200, 300, 400, 500}. As comparisons, we also consider existing atlases including the AAL (Rolls et al., 2020), Shen268 atlas (Shen et al., 2013), Shen368 atlas, and Schaefer atlas with400parcels (Schaefer et al., 2018). Each approach is evaluated under three states for the ABCD study (Rest, MID, nBack) and HCP study (Rest, Language, Emotion) to ensure reliability under different conditions. Given there is no tuning parameter involved in the existing atlases, when evaluating their connectivity-based prediction, we directly construct the region-wise connectivity matrix based on the specific atlas and apply it to each of the testing sets as described inSection 2.3.2. This ensures a fair comparison between our SBP and the existing ones with a consistent downstream prediction under CPM.

As a result, the out-of-sample R-squares along with standard deviation are presented inTable 1; and based on the results, we conclude that the proposed SBP significantly improves the predictive performance compared to the existing atlases across a range of region numbers. Notably, SBP shows the most marked improvement in predictive accuracy during resting conditions in both studies. For instance, in the ABCD study, the out-of-sample R-square increases by more than 170 times with the application of SBP compared with the existing ones. Moreover, the SBP consistently outperforms existing atlases across different task conditions, demonstrating its robust predictive capability through supervised connectivity biomarkers. An interesting observation from our comparison between the two studies is that SBP leads to a more pronounced prediction improvement in the ABCD study than in the HCP study. This discrepancy could be attributed to the children participants in the ABCD study. The fMRI scans of children often present more noise and exhibit stronger sample heterogeneity (Ota et al., 2014). These factors contribute to a more challenging analytical scenario, yet SBP successfully refines connectivity-based predictions with more informative functional parcellations that existing atlases struggle to achieve.

**Table 1.** Predictive performance as summarized by the out-of-sample$R^{2}$under SBP with different parcellation sizes and existing atlases for ABCD and HCP studies.

		ABCD			HCP		
Atlas # regions ( K )		Rest	MID	nBack	Rest	Language	Emotion
Proposed method							
SBP	100	0.064 (0.015)	0.086 (0.014)	0.078 (0.017)	0.045 (0.011)	0.117 (0.020)	0.109 (0.018)
SBP	200	0.075 (0.013)	0.105 (0.016)	0.094 (0.012)	0.058 (0.010)	0.124 (0.019)	0.119 (0.021)
SBP	300	0.088 (0.014)	0.106 (0.015)	0.103 (0.016)	0.062 (0.013)	0.132 (0.018)	0.131 (0.017)
SBP	400	0.092 (0.016)	0.112 (0.012)	**0.107 (0.011)**	**0.074 (0.013)**	**0.137 (0.014)**	**0.133 (0.015)**
SBP	500	**0.095 (0.012)**	**0.114 (0.010)**	0.103 (0.014)	0.071 (0.012)	0.135 (0.016)	0.129 (0.017)
SBP	600	0.093 (0.013)	0.110 (0.011)	0.105 (0.013)	0.070 (0.011)	0.133 (0.015)	0.130 (0.014)
Existing atlases							
AAL3	170	0.004 (0.007)	0.055 (0.009)	0.045 (0.008)	0.005 (0.006)	0.072 (0.011)	0.066 (0.010)
Shen268	268	0.008 (0.006)	0.072 (0.008)	0.066 (0.010)	0.012 (0.007)	0.106 (0.010)	0.093 (0.012)
Shen368	368	0.004 (0.005)	0.082 (0.009)	0.057 (0.007)	0.037 (0.008)	0.115 (0.012)	0.106 (0.011)
Schaefer	400	0.005 (0.007)	0.077 (0.010)	0.059 (0.009)	0.029 (0.008)	0.112 (0.011)	0.107 (0.013)

The standard deviation is provided in the parentheses. The best performance are shown in bold.

Finally, we observe that larger values ofK(400 and 500) generally yield better predictive performance compared to smaller ones. This suggests that when the network is too small, significant connections might be merged into single groups, potentially missing individual contributions. On the other hand, a network that is too large could lose its benefit by merging voxels into regions in terms of noise reduction and enhancement on interpretation. Additionally, both with our SBP method and other approaches, task-based fMRI shows higher predictive accuracy than resting-state data. This is in line with various previous studies (Chen et al., 2022;Greene et al., 2018) with task conditions showing a stronger connectivity-based predictive power. At last, to further benchmark the performance, we first implement SBP withλ=0as to an unsupervised algorithm to compare with our proposed method, where the detailed results along with statistical inference under Turkey’s HSD testing for between-method comparison are provided inSupplementary Table 1. Furthermore, we also implemented CPM with a lasso penalty for ABCD under MID task as an alternative to the ridge penalty. The results provided inSupplementary Table 3confirm that the superior predictive performance of SBP is robust and not dependent on the choice of regularization.

### 3.3 Supervised brain nodes

We then visualize our generated supervised brain nodes inTable 2with bothK=200(SBP(200)) and 400 (SBP(400)) under ABCD and HCP studies; and each node in the parcellation is color-coded. From left to right, the axial, sagittal, and coronal slices are visualized using Bioimage Suite (https://www.nitrc.org/projects/bioimagesuite/). As a direct consequence of assigning voxels based on their predictive power instead of solely on spatial proximity, the SBP-induced nodes exhibit higher variations across the whole brain. Unlike the AAL or Shen268 atlases, which are limited by fixed node boundaries, the SBP method dynamically generates atlases that are behavior-informed, state-specific, and trait-specific. Meanwhile, despite the different numbers of nodes, the generated parcellation schemes still demonstrate a certain degree of coherence. For instance, under the resting-state ABCD study, both SBP(200) and SBP(400) display large nodes in the inferior temporal gyrus as shown in the axial view. Across different states, larger nodes are noticeable in the temporal lobe, caudate nuclei, and hippocampus. We also calculate the ARI across different resolutions and cognitive states for SBP and compare these values with the ARI between SBP and existing atlases. As shown inSupplementary Table 2, the agreement between SBP at different resolutions is slightly higher than the agreement across different cognitive states. Both, however, show greater consistency than the comparisons between SBP and traditional static, unsupervised parcellations. Finally, being a whole-brain parcellation method, the SBP ensures a degree of spatial contiguity through the adjacency matrix, as shown in the obtained brain nodes. Under different states, with distinct predictive roles that functional signals involved, our constructed parcellations are also thoroughly tailored.

**Table 2.** Supervised brain nodes generated with SBP(200) and SBP(400) for intelligence scores under ABCD and HCP studies.

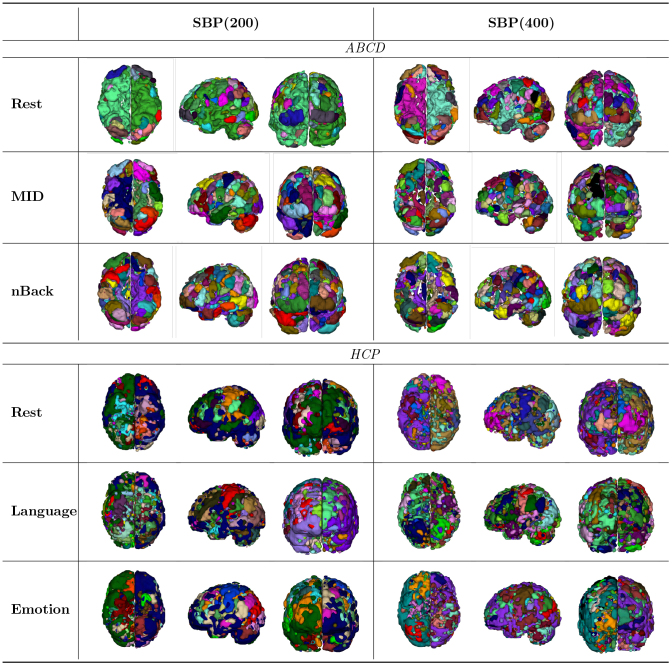

### 3.4 Functional network at large-scale network level

Having established that the proposed SBP consistently enhances predictive accuracy for new samples, we further explore the reasons behind this improved performance and the established functional signals linked to the targeted outcome. We consider the parcellations generated from SBP(400) as an illustrative example, given its consistently superior performance across ABCD and HCP studies and the comparable number of nodes with existing atlases. For each state and each study, we focus on the top20%functional connections identified by the last step CPM, and map them to the ten canonical neural networks (Yeo et al., 2011) as shown inFigure 4. In each subfigure of our analysis, we present heatmaps that show the number of these functional connections, both within and between the canonical neural networks. We separately summarize the heatmaps by distinguishing between connections that are positively and negatively associated with the outcome.

**Fig. 4.**
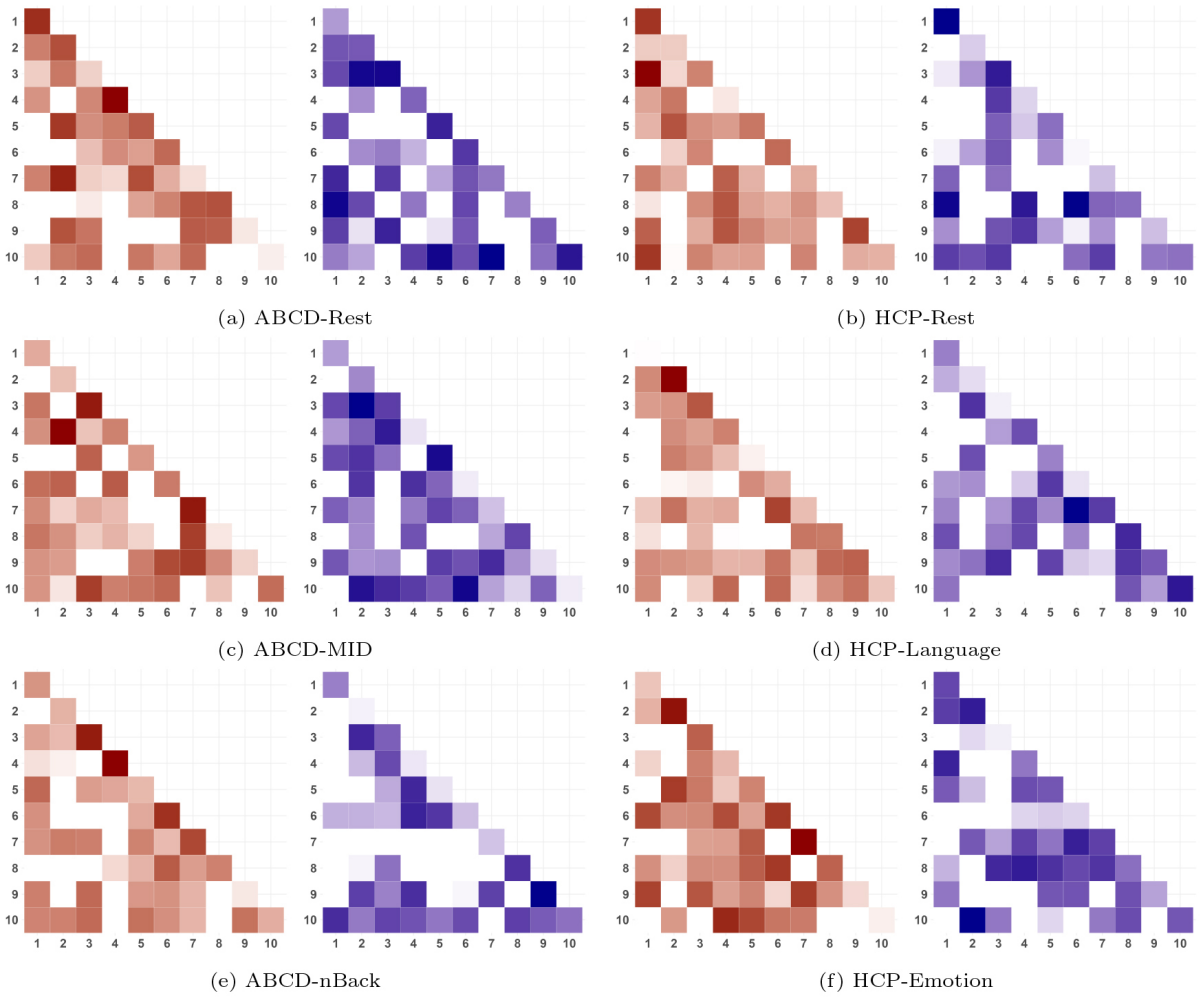
Heatmaps summarize the number of the top20%selected connections with the highest absolute value of coefficient from CPM based on SBP(400) parcellation. The left and right column correspond to the ABCD study under resting (a), MID (c) and nBack (e) tasks, and HCP study under resting (b), Language (d) and Emotion (f) tasks, respectively, and each row corresponds to a specific state. Within each subfigure, the connections with positive coefficients are colored red, and the negative coefficients are colored blue. The canonical neural networks in the plots correspond to: 1. medial frontal, 2. fronto parietal, 3. default mode, 4. motor, 5. visual I, 6. visual II, 7. visual association, 8. limbic, 9. basal ganglia, and 10. cerebellum.

The observed patterns in our analysis indicate that our parcellation schemes are effective at identifying both inter-network connections and intra-network ones, and the informative network neuromarkers vary across different states and study populations. In the ABCD study, the motor and default mode networks heavily contribute to both inter- and intra-network connectivity neuromarkers in positive networks across states; and the cerebellum network also plays a role on top of aforementioned networks in negative networks. In the HCP study, the medial frontal network is highlighted to offer inter- and intra-network connections under resting-state, while the fronto parietal network contributes substantially under task conditions along with the cerebellum. To further explore differences in brain functional signal distribution between the two studies, we present the absolute percentage difference in the number of behaviorally relevant functional connections within and between canonical neural networks inSupplementary Figure 1. Although the overall differences within and between network systems remain relatively small, more pronounced differences are observed in the medial frontal, basal ganglia, and cerebellar networks. These findings may reflect developmental differences in how brain networks contribute to behavior, with distinct patterns of functional involvement between preadolescents in the ABCD study and young adults in the HCP study.

Furthermore, we examine the canonical neural network distribution of the top 5 largest nodes identified by SBP(400) under both studies, presented inFigure 5. The results indicate a diverse functional network composition within these top nodes, and the architectural patterns also vary across different cognitive conditions and studies. For example, in the resting state of the ABCD study, the second-largest node contains13.56%of its voxels from the cerebellum network (highlighted in yellow), which represents the largest percentage of cerebellum among the top five nodes. During the MID and nBack tasks, node 5 and node 3 comprise19.06%and21.13%of voxels within the cerebellum network, respectively. We also observe that the proportion of Visual II (indicated in purple) is significantly lower in general under the nBack task compared to the resting state. In the HCP study, node 3 in the emotion task comprises18.65%of its voxels within the basal ganglia (marked in teal) network, whereas the largest node from the Language task contains only11.86%. Such diversity in patterns and network compositions highlights the supervised nature of our brain parcellation approach. Unlike existing atlas methodologies that often strive for intra-node functional homogeneity, our method acknowledges and integrates the functional diversity within each node to enhance the predictive power for behavior outcomes.

**Fig. 5.**
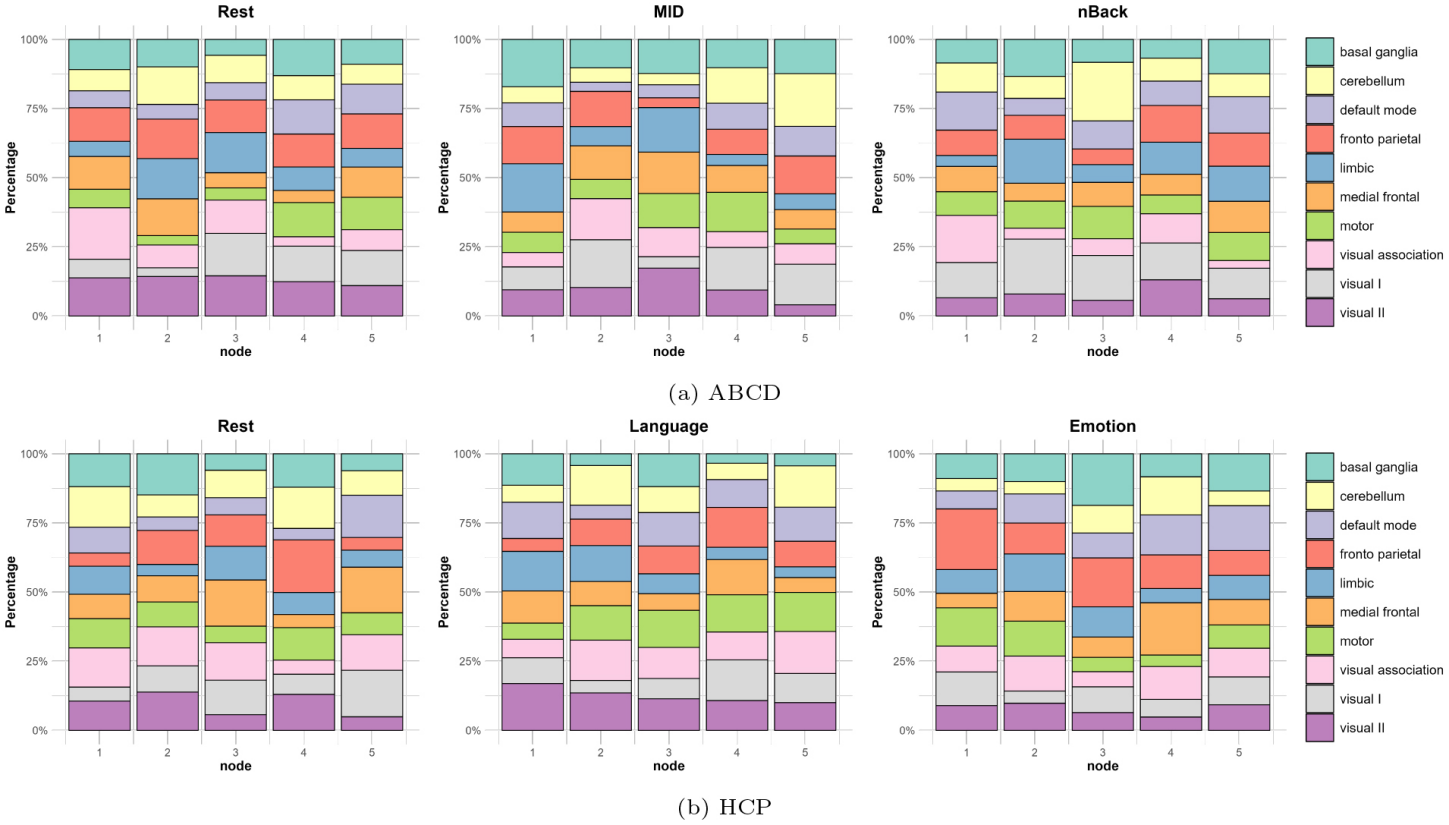
Distribution among canonical neural networks for the largest five nodes constructed by SBP(400) for (a) ABCD study and (b) HCP study.

### 3.5 Functional network anatomy

To illustrate the informative network configurations constructed by SBP(400) in relation to the macroscale brain structure, we focus on the informative functional connections with the outcome under a correlation strength larger than 0.1 for each state and each study. These informative networks are displayed inFigure 6, where we separately present positive and negative correlated connections. In each subfigure, nodes are arranged in two semicircles, approximating the brain’s anatomy from anterior (top of the circle, 12 o’clock position) to posterior (bottom of the circle, 6 o’clock position), color-coded according to cortical lobes. The nodes (inner circle) are anatomically grouped into lobes (outer circle) split into left and right hemispheres with each line representing an informative connection.

**Fig. 6.**
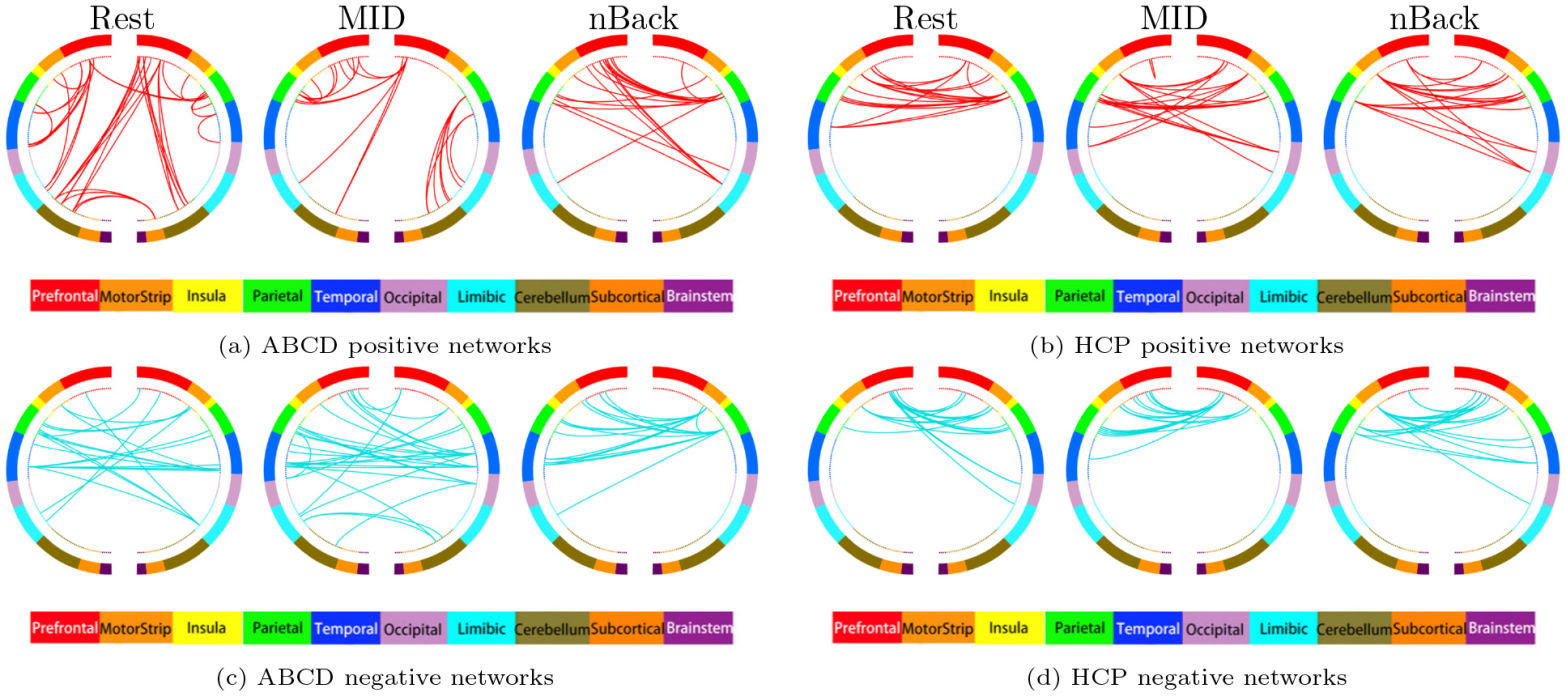
A circular graph represents the significant positive and negative functional networks. Macroscale brain regions are color-coded as in the legend, and the cyan lines represent the significant connections. Subfigures (a), (b), (c), and (d) are the positive and negative networks for ABCD and HCP studies, respectively.

Based on the result, similar to previously identified connectivity features (e.g.,Finn et al. (2015)), the behavior-related network configurations are generally complex and span across various brain macroscale areas. In each state and each study, we observe a large proportion of long-range, cross-hemisphere connections in both positive and negative networks, which demonstrates their dominant predictive role for general cognitive ability. Among those connections, many are between different lobes or occur within a single lobe but across hemispheres. For instance, the functional networks linking parietal and temporal lobes are particularly prominent in resting and nBack states for the ABCD study, and in the resting and language states for the HCP study. The connections between the parietal and motor are also extensively observed in most conditions under both studies. Furthermore, there are a higher number of informative connections in certain areas such as prefrontal, motor, and parietal involved compared to the occipital, cerebellum, and subcortical lobes, especially during tasks. Finally, we repeat the analysis under each resting-state session of HCP, and calculate the correlation between the number of significant connections within and between functional systems across the two sessions. This analysis yielded a high correlation of 0.92 with positive and negative correlated connections under each session shown inSupplementary Figure 4, demonstrating a reproducibility of the SBP-derived functional connections.

### 3.6 Reproducibility

In this section, we evaluate the reproducibility of the supervised brain nodes generated by SBP among different runs. As detailed inSection 2.4, under each of the 20 random samples, we evaluate the robustness of the node size by calculating the number of voxels in each node, and summarizing the distributions of its averaged values across all the nodes under each state and study as shown in the upper left panel ofFigure 7. The results demonstrate that the parcellation procedure consistently produces comparable node sizes under each cognitive state, eachKvalue, and each study. Regarding the parcellation agreement, the distribution of ARI values, presented in the upper right panel ofFigure 7, and the Dice coefficients, shown inSupplementary Figure 2, provide insights into the agreement of node parcellation across different runs. Notably, the ARI values indicate moderate reproducibility, while the Dice coefficients are slightly lower. This is anticipated given the calculation involves aligning parcellations from different random samples, a process that can be challenging due to the flexible nature of node definitions in our method. While this flexibility may lead to some degree of loss in reproducibility, these values still suggest a reasonable level of consistency in the parcellation outcomes across conditions and studies despite variability in sample composition. Finally, to further demonstrate the findings inFigure 7a-b, we show that the results go beyond what is expected under the null hypothesis generated by randomly dividing the brain intoKparcels. We compare the reproducibility measured by the Dice coefficient between SBP and both spectral clustering and a random baseline parcellation under the ABCD dataset withK=200. For the random baseline,Kvoxels were randomly selected and then expanded to cover the entire brain. As shown inFigure 7c, SBP achieves significantly higher reproducibility than both spectral clustering and random parcellation, demonstrating its robustness.

**Fig. 7.**
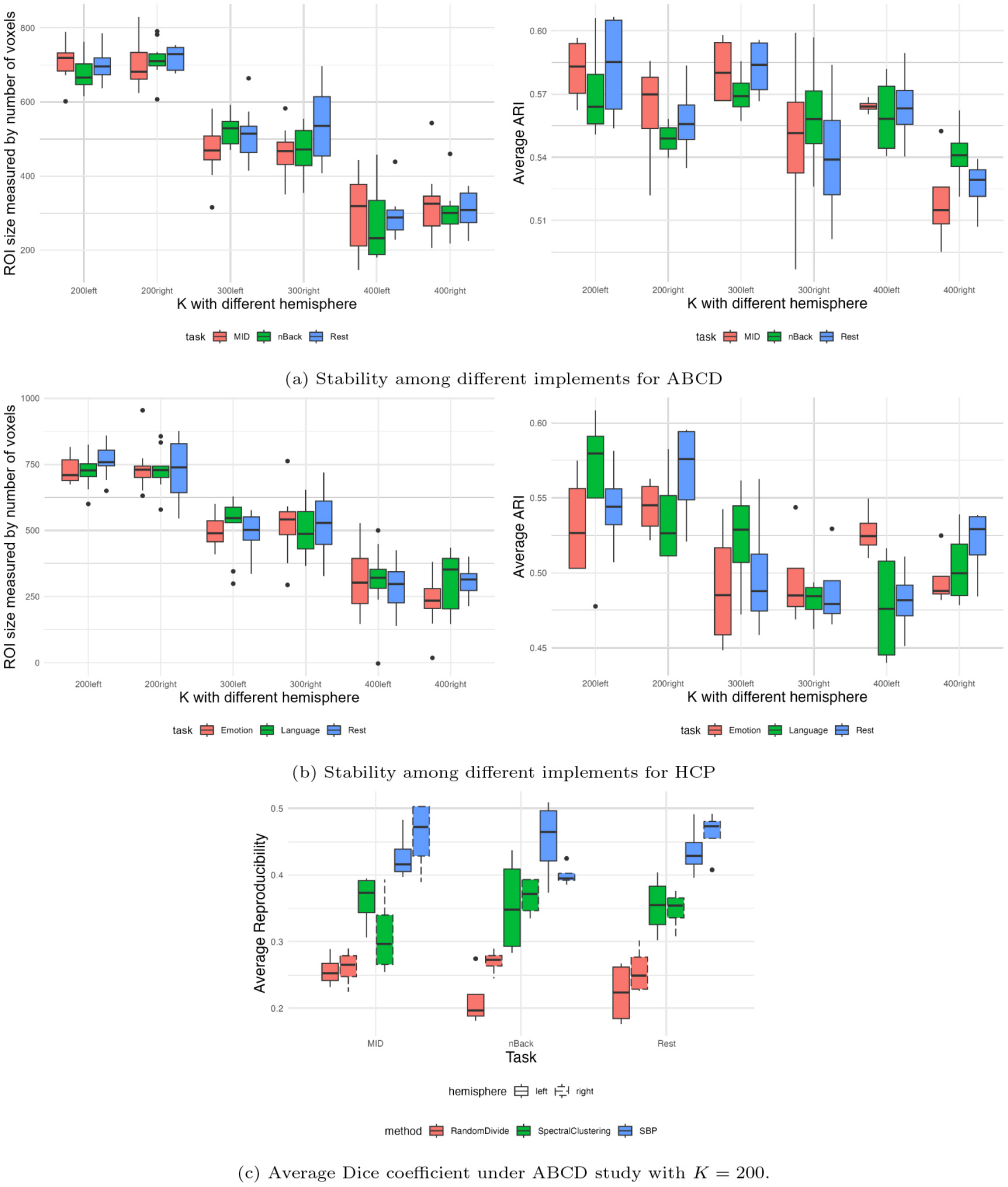
Node size stabilization and average ARI for (a) ABCD and (b) HCP under differentKand states. (c) Average Dice coefficient under ABCD study withK=200.

## 4 Discussion

In this work, we introduce the SBP framework, a novel approach designed to meet the urgent demand for more informative functional brain parcellations, particularly in the context of predictive tasks. Our framework uniquely defines brain functional nodes and their networks, taking into account their relationship with relevant behavioral outcomes. Our analytical framework is built under a regularized spectral clustering algorithm, which facilitates graph-based community detection while integrating behavioral outcome associations through a separation preference matrix. The extensive numerical analyses, using both simulated data and two landmark multi-state fMRI studies, consistently highlight the superior performance of SBP in enhancing connectome-based predictions. This advancement not only outperforms predictions based on existing brain atlases but also lays the groundwork for establishing behavior-refined and more informative functional brain nodes and networks. It is also worth noting that our node construction procedure is impacted by co-activation information, while simultaneously penalized by predictive relevance. Thus, our nodes cannot be interpreted simply as functionally modular regions, or anatomically modular regions. These regions are best understood as*predictive brain regions*—clusters of voxels that share a consistent relationship with behavioral outcomes rather than purely reflecting functional or anatomical modularity. Unlike traditional parcellation methods that prioritize anatomical or functional modularity, SBP is designed to balance functional co-activation patterns with their relevance to behavioral prediction. This allows the resulting parcels to be behaviorally coherent with respect to a behavior outcome.

Our optimization function currently integrates both an unsupervised spectral learning of connectivity data and a separation procedure guided by the behavior outcome. The spectral learning component inherently promotes some spatial smoothness. However, with an emphasis on the behavior-guided component, a single node could separate into non-contiguous segments, without a guarantee of spatial continuity. While traditional brain atlases focus on spatially contiguous regions, such contiguity is not considered to be a strict requirement. Nevertheless, spatially contiguous functional nodes do offer enhanced interpretability. The spatial contiguity in our method is inherently incorporated through the spectral clustering term, which leverages the low-dimensional structure learned from the voxel-level adjacency matrix. This adjacency matrix, derived from functional connectivity data, captures the spatial organization of voxels and their intrinsic relationships. Given the inherently smooth nature of fMRI signals at the voxel level, the learned low-dimensional representation promotes spatially contiguous clustering of functionally similar voxels. This tendency toward spatial contiguity is consistent with findings from previous studies, includingShen et al. (2013). However, our learning framework is designed to balance spectral clustering with behavioral relevance regularization. As a result, spatial contiguity is encouraged but not strictly enforced. The final optimization reflects a trade-off between these two objectives.

In light of this, a potential improvement to our SBP framework could involve the integration of spatial information into the objective function via a spatial regularization term. Such an enhancement would not only preserve the method’s predictive power but also improve its anatomical interpretability. Although the proposed SBP method is fundamentally a group-level parcellation scheme leveraging the brain-to-behavior associations within a cohort, it can be readily applicable to studies with multiple subgroups. The current aggregation for adjacency matrices, which is on a single group setting, can be extended to construct group-specific adjacency matrix for each subgroup. This would allow us to create distinct parcellations tailored to the unique functional connectivity patterns within each subgroup. Additionally, we are interested in investigating as future studies how the parcellation changes across subgroups and whether certain patterns are consistent or distinct between them.

Another potential extension is on the construction of both the connectivity adjacency matrixAand the regularization matrixR. Currently, both matrices rely on Pearson correlation for measuring voxel-wise similarity and edge-behavior association, respectively. While Pearson correlation offers straightforward numerical operation and interpretation, it primarily captures linear relationships, potentially overlooking complex higher-order and nonlinear correspondences. Therefore, to address this potential limitation, kernel-based or neural-network-based approaches could be developed as alternative options to provide more nuanced characterizations of those complex relationships. Furthermore, we currently focus on predicting the general cognitive score as a canonical behavior outcome to demonstrate the efficacy of our framework. Going forward, it would be beneficial to broaden our scope to include other behavioral traits and disease profiles to establish more tailored functional nodes and networks that are relevant to a wider array of behavioral and clinical contexts.

## Supplementary Material

Supplementary Material

## Data Availability

This work adopted publicly available data from the ABCD and HCP studies. The ABCD data are available through the NIMH Data Archive (NDA).https://nda.nih.gov/, and the HCP data can be accessed via ConnectomeDB (https://db.humanconnectome.org). The code for the proposed SBP algorithm is available athttps://github.com/wanwanx/SBP.
