# Supplementary material for: Supervised brain node and network construction under voxel-level functional imaging
Source: Imaging Neurosci (Camb). 2025 Jun 26;3:IMAG.a.56. doi: 10.1162/IMAG.a.56 (PMC12319940; doi:10.1162/IMAG.a.56)
Supplement: Supplementary Material [file imag.a.56_supp.pdf]

Suppmentary materials for “Supervised brain node and network construction under voxel-level functional imaging”

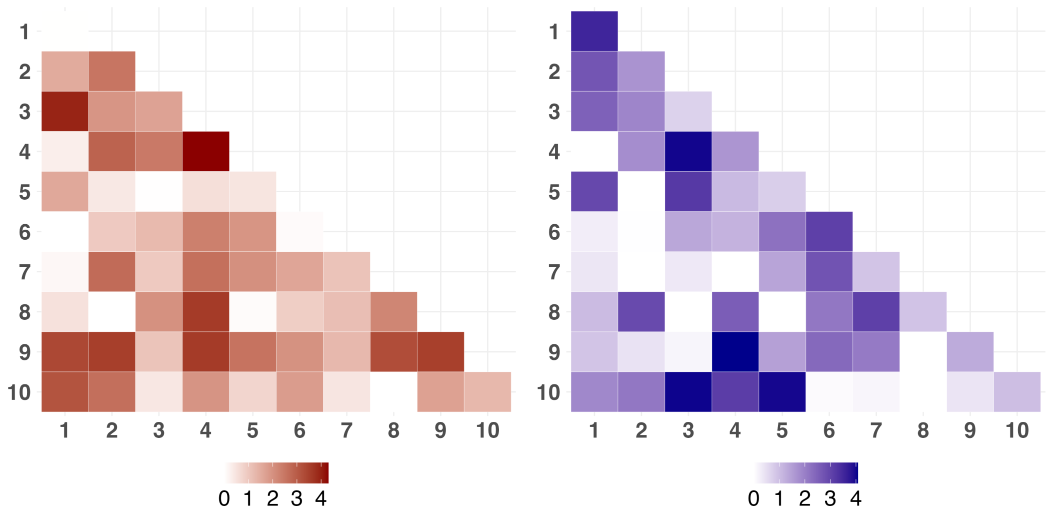

Supplementary Figure 1: The absolute percentage difference between ABCD and HCP selected resting-state functional connections is shown. The left panel displays positive coefficients, while the right panel shows negative coefficients. The canonical neural networks in the plots correspond to: 1. medial frontal, 2. fronto-parietal, 3. default mode, 4. motor, 5. visual I, 6. visual II, 7. visual association, 8. limbic, 9. basal ganglia, and 10. cerebellum.

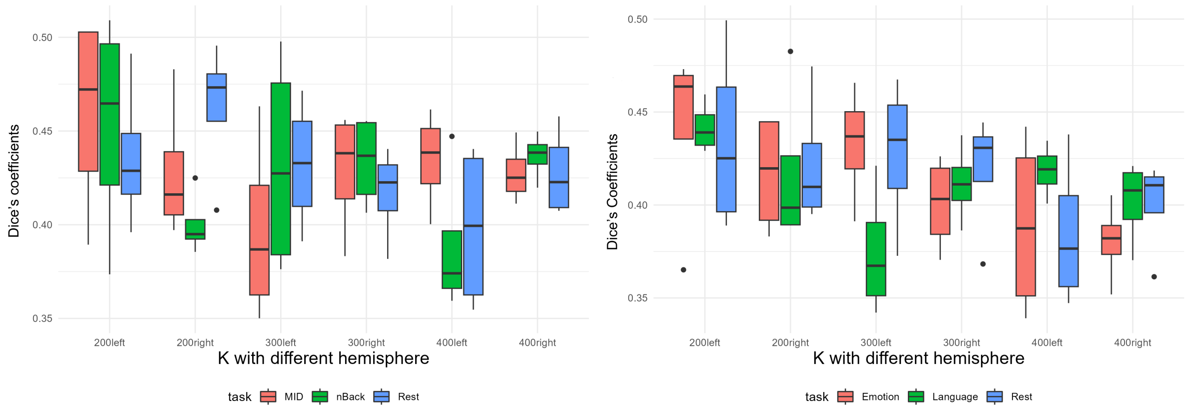

Supplementary Figure 2: Stability by the Dice’s Coefficient among different implements for ABCD and HCP.

Supplementary Table 1: Predictive performance and comparisons among methods under Turkey's test.

Atlas	# Regions (K)	ABCD			HCP		
		Rest	MID	nBack	Rest	Language	Emotion
Proposed method							
SBP	100	0.064 (0.015)	0.086 (0.014)	0.078 (0.017)	0.045 (0.011)	0.117 (0.020)	0.109 (0.018)
SBP ($\lambda = 0$)	100	0.006 (0.005)	0.065 (0.008)	0.053 (0.010)	0.022 (0.007)	0.095 (0.010)	0.085 (0.011)
SBP	200	0.075 (0.013)	0.105 (0.016)	0.094 (0.012)	0.058 (0.010)	0.124 (0.019)	0.119 (0.021)
SBP ($\lambda = 0$)	200	0.008 (0.006)	0.074 (0.009)	0.059 (0.011)	0.035 (0.008)	0.101 (0.012)	0.089 (0.010)
SBP	300	0.088 (0.014)	0.106 (0.015)	0.103 (0.016)	0.062 (0.013)	0.132 (0.018)	0.131 (0.017)
SBP ($\lambda = 0$)	300	0.010 (0.006)	0.080 (0.007)	0.062 (0.009)	0.039 (0.009)	0.110 (0.011)	0.095 (0.012)
SBP	400	0.092 (0.016)	0.112 (0.012)	**0.107 (0.011)**	**0.074 (0.013)**	**0.137 (0.014)**	**0.133 (0.015)**
SBP ($\lambda = 0$)	400	0.012 (0.007)	0.083 (0.008)	0.065 (0.010)	0.041 (0.010)	0.113 (0.013)	0.099 (0.011)
SBP	500	**0.095 (0.012)**	**0.114 (0.010)**	0.103 (0.014)	0.071 (0.012)	0.135 (0.016)	0.129 (0.017)
SBP ($\lambda = 0$)	500	0.013 (0.007)	0.085 (0.009)	0.066 (0.008)	0.043 (0.009)	0.114 (0.012)	0.101 (0.013)
SBP	600	0.093 (0.013)	0.110 (0.011)	0.105 (0.013)	0.070 (0.011)	0.133 (0.015)	0.130 (0.014)
SBP ($\lambda = 0$)	600	0.011 (0.005)	0.080 (0.011)	0.068 (0.009)	0.039 (0.007)	0.112 (0.009)	0.117 (0.014)
Existing atlases							
AAL3	170	0.004 (0.007)	0.055 (0.009)	0.045 (0.008)	0.005 (0.006)	0.072 (0.011)	0.066 (0.010)
Shen268	268	0.008 (0.006)	0.072 (0.008)	0.066 (0.010)	0.012 (0.007)	0.106 (0.010)	0.093 (0.012)
Shen368	368	0.004 (0.005)	0.082 (0.009)	0.057 (0.007)	0.037 (0.008)	0.115 (0.012)	0.106 (0.011)
Schaefer	400	0.005 (0.007)	0.077 (0.010)	0.059 (0.009)	0.029 (0.008)	0.112 (0.011)	0.107 (0.013)
Difference in prediction (pvalue) between methods							
Spectral Cluster(400) vs. SBP(400)		-0.079 (0.000)	-0.029 (0.000)	-0.040 (0.000)	- 0.033 (0.000)	-0.024 (0.003)	-0.038 (0.003)
AAL3 vs. SBP(400)		-0.088 (0.000)	-0.057 (0.000)	-0.062 (0.000)	-0.069 (0.000)	-0.065 (0.000)	-0.067 (0.000)
Shen268 vs. SBP(400)		-0.082 (0.000)	-0.040 (0.000)	-0.041 (0.000)	-0.062 (0.000)	-0.031 (0.000)	-0.040 (0.000)
Shen368 vs. SBP(400)		-0.088 (0.000)	-0.040 (0.000)	-0.050 (0.000)	-0.037 (0.000)	-0.022 (0.001)	-0.027 (0.000)
Schaefer vs. SBP(400)		-0.087 (0.000)	-0.035 (0.000)	-0.048 (0.000)	-0.045 (0.000)	-0.025 (0.002)	-0.026 (0.002)

Supplementary Table 2: Adjusted rank index for SBP under different resolutions and states compared with existing atlases.

	ABCD				HCP			
	Rest-MID	Rest-nBack	Rest-AAL	Rest-Shen268	Rest-Language	Rest-Emotion	Rest-AAL	Rest-Shen268
SBP(200)	0.5837	0.5321	0.3704	0.3516	0.5280	0.5398	0.3449	0.3842
SBP(300)	0.4957	0.5201	0.3610	0.3863	0.4716	0.4803	0.3631	0.3795
SBP(400)	0.4787	0.4902	0.3447	0.3212	0.4119	0.4053	0.3380	0.3561
	SBP(300)	SBP(400)			SBP(300)	SBP(400)		
SBP(200)	0.6624	0.6315			0.6176	0.5832		

Supplementary Table 3: Predictive performance of SBP parcellations with Ridge-CPM and Lasso-CPM.

	SBP(100)	SBP(200)	SBP(300)	SBP(400)	SBP(500)	SBP(600)
Ridge-CPM	0.086 (0.014)	0.105 (0.016)	0.106 (0.015)	0.112 (0.012)	0.114 (0.010)	0.110 (0.011)
Lasso-CPM	0.079 (0.011)	0.106 (0.007)	0.108 (0.013)	0.111 (0.009)	0.112 (0.010)	0.009 (0.008)

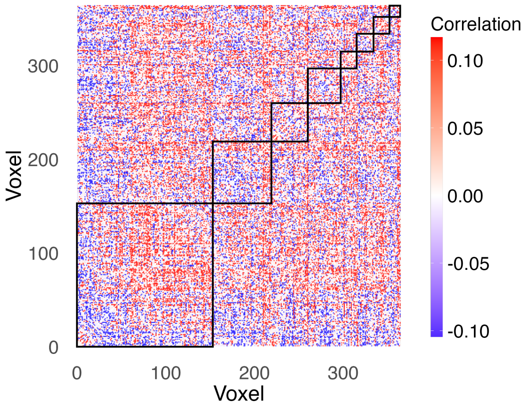

(a) SBP(200)

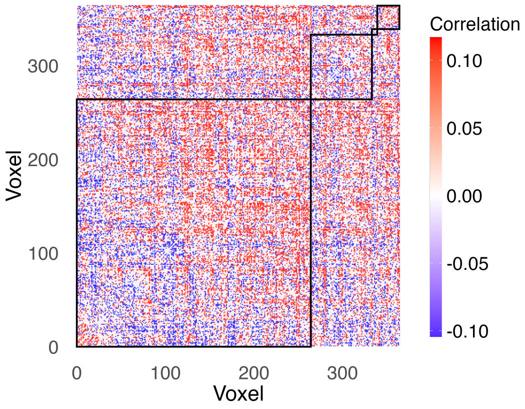

(b) Spectral clustering (200)

Supplement Figure 3: Heatmap of functional connectivity among voxels in node 184 (left parietal area) from the Shen268 atlas. Black lines delineate the identified nodes obtained using the SBP algorithm (left) and spectral clustering (right). The data was randomly split into two halves, with one used to train SBP and the other to train spectral clustering at $K = 200$. In the figure below, voxels corresponding to the original node 184 were identified and mapped to their respective nodes under SBP (left) and spectral clustering (right). The results show that SBP provides finer parcellation, which could effectively capture heterogeneous intra-node signals. However, it is important to note that SBP is designed to optimize behaviorally significant connections by promoting them as inter-node connections. As a result, visualizing parcellation boundaries which may be more relevant to reflect intra-node coherence may not fully reflect SBP's advantages. Instead, we consider that our numerical studies have provided strong evidence, confirming that SBP significantly outperforms spectral clustering in predictive power.

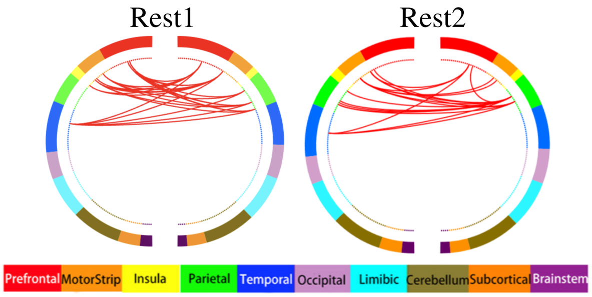

(a) HCP positive networks

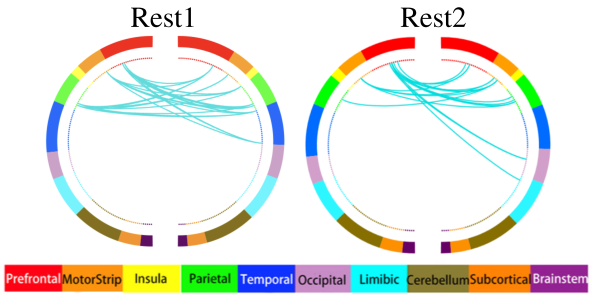

(b) HCP negative networks

Supplement Figure 4: A circular graph represents the significant positive and negative functional networks. Macroscale brain regions are color-coded as in the legend, and the cyan lines represent the significant connections. Subfigures (a) and (b) are the positive and negative networks for the two HCP resting sessions, respectively.
